# Topical betamethasone and systemic colchicine for treatment of recurrent aphthous stomatitis: a randomised clinical trial

**DOI:** 10.1186/s12903-023-03335-x

**Published:** 2023-10-03

**Authors:** Surab Alsahaf, Khlood A. Alkurdi, Stephen J. Challacombe, Anwar R. Tappuni

**Affiliations:** 1 Oral Medicine, Centre for Host Microbiome Interactions, Faculty of Dentistry, Oral & Craniofacial Sciences, King’s College London, and Guys and St Thomas’ NHS Foundation Trust, London, UK; 2 Institute of Dentistry, Faculty of Medicine and Dentistry, Queen Mary University of London, Office 7, Floor 4, London, E1 2AD UK

**Keywords:** Oral ulcers, Ulcer severity, Clinical outcomes, Betamethasone, Colchicine

## Abstract

**Background:**

Recurrent Aphthous Stomatitis (RAS) is painful oral ulceration frequently treated with topical steroids. There is limited published evidence for the efficacy of any treatment for RAS and there remains a need for longitudinal randomised clinical trials to evaluate and compare the effectiveness of different therapies in the management of RAS. The aim of the current project was to assess the efficacy of betamethasone mouthwash and colchicine tablets, individually and combined, for the treatment of RAS, and to establish the optimum treatment period necessary for a significant reduction in the disease severity.

**Methodology:**

A randomised, prospective, parallel-group clinical trial was conducted over one year, to compare the efficacy of three therapies in RAS. One hundred and six patients were randomized into three groups; 35 received betamethasone mouthwash, 35 had colchicine tablets and 36 received both therapies. The response was evaluated quantitatively every 3 months for 1 year, using the Ulcer Severity Score (USS).

**Results:**

For all three treatment regimes, the mean USS decreased by about 30% in the first 3 months (p < 0.001). Further improvement was noted for up to 9 months. At the end of the study, the mean USS had improved by 50% from 34.9 ± 7.2 before treatment to 17.5 ± 8.9 after treatment (p < 0.001). Of included participants, 86% showed significant clinical improvement by the end of the study. There were no significant differences in outcomes between the three regimes (p < 0.05).

**Conclusions:**

This clinical trial has provided evidence for the efficacy of betamethasone mouthwash and for colchicine tablets in the treatment of RAS and has shown that at least six months of treatment may be required for optimum effect.

**Clinical trial registration number:**

ISRCTN3267716. **Date of clinical trial registration:** 15/04/2018

## Background

Recurrent Aphthous Stomatitis (RAS) is a relatively common oral mucosal condition affecting about 10% of the population with a varied reported prevalence of 1.5-25% [1, 2]. RAS is a chronic condition that usually starts in teenage years, with various degrees of severity [3]. Despite vast clinical experience in managing this condition, there is little clinical evidence supporting current therapies, which remain largely palliative [4].

Topical corticosteroids are the mainstay of RAS treatment [4, 5]. In specialised oral medicine clinics in the UK, betamethasone sodium phosphate mouthwash, a steroid used to treat other conditions such as Rheumatic Diseases, is used (off licence) as the first line treatment for RAS, but there is limited published evidence for its efficacy [6]. Therefore, there is a need for longitudinal randomised clinical trials to evaluate the efficacy of betamethasone mouthwash for the management of RAS, and for comparison with other treatment regimes.

Colchicine, is an anti-inflammatory agent with some immunosuppressive action, used for treating gout and other conditions including PFAPA syndrome and Behçet’s Syndrome [7–9]. Colchicine therapy has been used in specialised clinics for the more severe cases of RAS [10, 11] but systematic reviews have concluded that further clinical trials are needed [12]. Some studies have supported the efficacy and safety of colchicine for controlling RAS [6, 13], but none were formal comparisons with other treatment modalities.

Evidence based clinical research requires disease severity assessment and outcome measures. In recent years, oral disease severity scoring systems [14], including ulcer severity scores [6], provided tools for this assessment, and their use in trials is increasing [15]. The aims of the current study were to provide clinical evidence for the efficacy of betamethasone mouthwash in the treatment of RAS and to compare it to the efficacy of systemic colchicine, and that of dual therapy of both betamethasone and colchicine. A secondary aim was to determine the optimal duration of treatment to achieve a significant reduction in the ulcer severity score (USS). Previous studies have indicated that a USS of 20 or less could be classified as mild and that this was a threshold for deciding whether active treatment of any kind was warranted [6]. The therapy regimes study used in this study were according to the routine clinical practice in our oral medicine clinics for the management of RAS.

## Study design

A randomised, prospective, parallel-group, single-centre clinical trial was designed to investigate the efficacy of three different modalities of treatment: betamethasone mouthwash, colchicine tablets, and a combination of the two. The trial was conducted in 12 month period and in compliance with the principles of the Declaration of Helsinki (2013), the principles of International Council for Harmonisation- Good Clinical Practice (ICH-GCP), and all the applicable regulatory requirements. The study was approved by the Guy’s Research Ethics Committee and the Medicines and Healthcare Products Regulatory Agency. This clinical trial was fully registered with The International Standard Randomised Clinical Trial Number (ISRCTN). Written informed consent was obtained from all participants.

### Study subjects

#### Study groups

Two hundred and one patients with RAS were screened and 95 were excluded as they did not meet the inclusion criteria (Table 1). Screening included haematological investigations for haematinics, renal and liver serology. A number of 106 RAS patients were randomly assigned to one of the three treatment groups (see below) and assessed at 3 months intervals for 12 months. A total of 46 males aged 21–63 years (mean 39.2 years) and 60 females aged 22–65 years (mean of 40 years) were included. In 51 subjects, RAS was of the minor type, while 53 had major and only 2 participants had herpetiform RAS.

#### Group 1

Thirty-five subjects were prescribed betamethasone sodium phosphate mouthwash made up by dissolving a 500 mcg tablet in 10ml of water and used as a mouthrinse for 3 min then discarded. It was administered four times a day when ulcers are present and twice a day in between ulcer attacks.

#### Group 2

Thirty-five subjects were prescribed colchicine 500 mcg tablets to be taken once daily after breakfast.

#### Group 3

Thirty-six subjects were prescribed colchicine 500 mcg tablets once daily and betamethasone sodium phosphate 500 mcg tablet dissolved in 10 ml of water and used as a mouthrinse for 3 min then discarded, four times a day during ulcer attacks only.

**Table 1:** Participant inclusion and exclusion criteria

Inclusion criteria	Exclusion criteria
Diagnosed with RAS according to the original criteria of Lehner (1968).	Unwilling or unable to comply with the study protocol.
Aged between 18–65 years.	Pregnant or breast feeding.
Willing and able to give informed consent.	Atopic or who had a relevant history of allergy, hypersensitivity or side effects or contraindications to any of the study medications.
Not involved in other studies that would compromise their safety or undermine the scientific basis of the study.	Diagnosed with systemic diseases thought to overlap with RAS.
Free of any known co-morbidities.	Treated with local or systemic steroids or colchicine within the previous three months
-	Patients with an USS score of less than 20.

### Randomisation

**Fig. 1:**
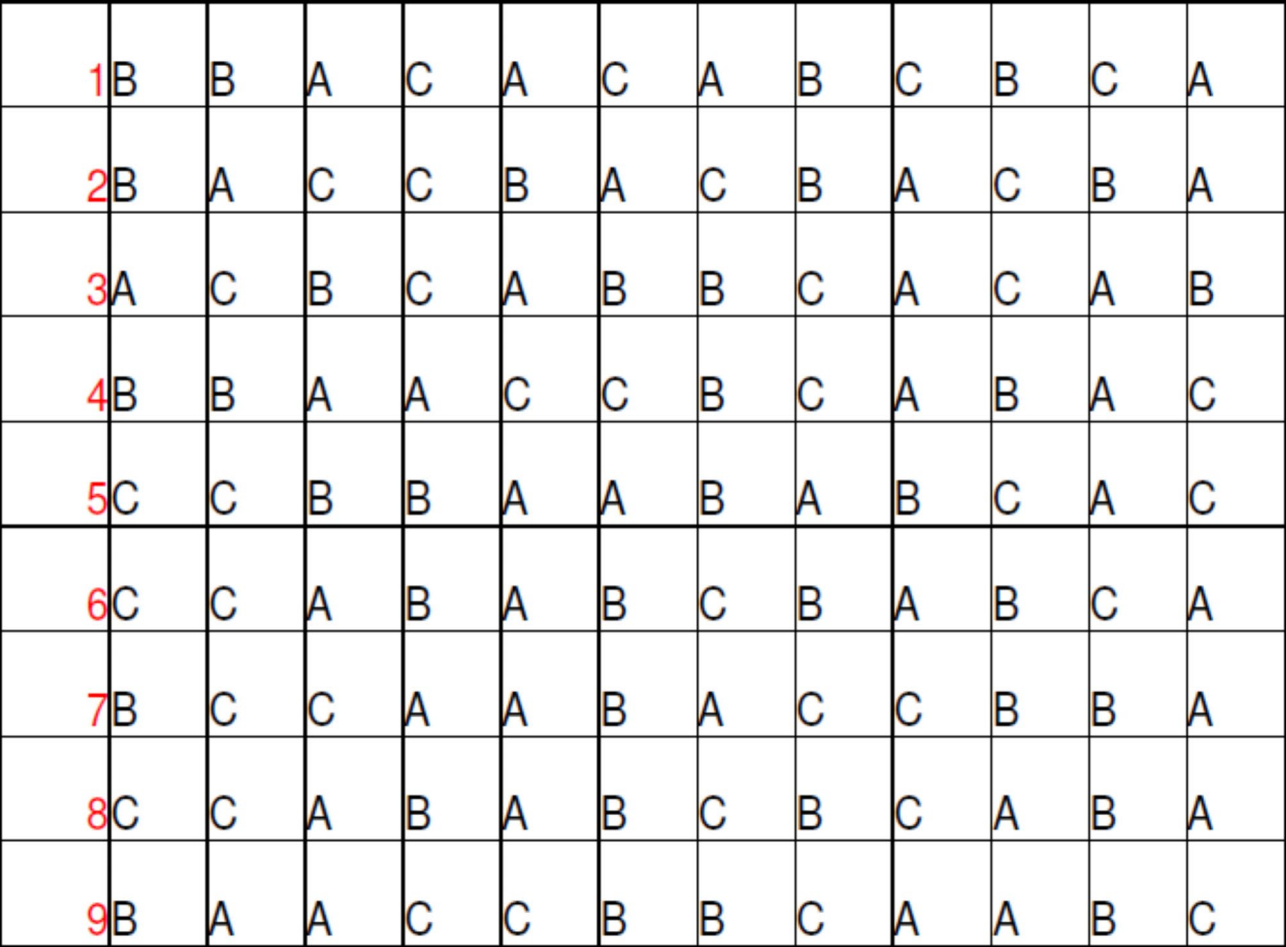
Randomization table following the sequence of “ABC” where **(A)** is Colchicine tablet and Betnesol mouthwash, **(B)** is Colchicine tablet and **(C)** is Betnesol mouthwash

A table of randomised numbers was used in this study in the sequence of “ABC” where (A) is Colchicine tablet and Betnesol mouthwash, (B) is Colchicine tablet and (C) is Betnesol mouthwash (Fig. 1). In addition, a research nurse was in charge of the participants’ randomization, their assignments to the study groups and their review appointments. However, the sequence was concealed until the effect of all medications were analyzed.

### Assessment of Ulcers

**Fig. 2:**
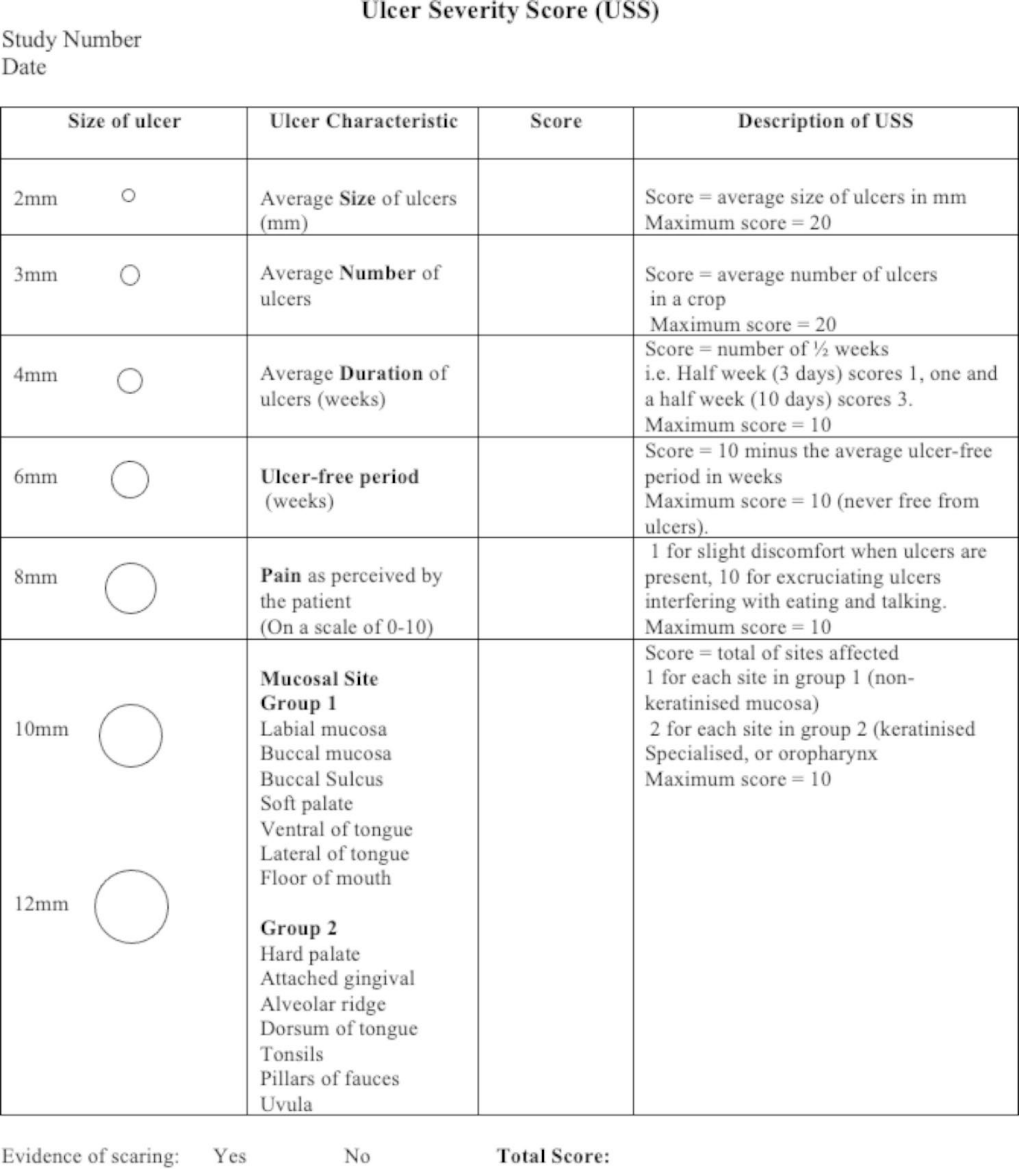
Form used for the routine assessment of the Ulcer Severity Score (USS). Modified from Tappuni et al. (2013) [6]. The total USS is the sum of five objective measurements and one subjective (pain) from recent clinical history added to a pain score

Ulcers severity was assessed using the USS, first described by Tappuni et al. in 2013^6^. At each visit, the characteristics of the ulcer attacks in the preceding three months were recorded on a standardised form (Fig. 2). The patients kept a diary of the ulcers; average size, number of ulcers, duration, frequency of ulcer attacks, sites affected, and the intensity of the pain caused by the ulcers. This information was verified clinically whenever possible. An USS form was completed on each study visit, and the ulcers severity was scored independently without referring to the score of previous visits. The USS was expressed as a numerical score, which enabled an objective comparison of the severity of the condition before and after treatment [6].

### Study visits

#### Baseline visit

Patients meeting the inclusion criteria were randomised by the clinical trial pharmacist at Guy’s Hospital and assigned to one of the three treatment groups described above. Clinicians were not involved in the randomisation or the treatment assignment, and thus were blinded to the treatment taken for the first 3 months of the study. However, due to the different mode of administration of the study medications (as colchicine is a tablet and betamethasone is a mouthwash), neither the participants nor the clinical examiner was blinded further than the first 3 months of the trial. Patients were given a standardized diary and shown how to perform self-examination for the recognition of oral ulcers as previously described.

### Treatment visits

Participants were assessed at 3-month intervals for a year (four visits). At each visit, the same examiner evaluated the information recorded in the diary and validated it by history taking before calculating the USS. To minimise bias, the recording and the calculation of the USS was carried out independently of the findings at previous visits, and all participants were assessed twice by the same two clinicians on all visits. The remaining medication tablets were counted at each visit, to assess participants’ compliance with the treatment regime. Those who were deemed non-compliant were excluded from the study. At each visit, blood tests for haematinics, liver and kidney function, blood pressure and weight recordings were all performed to monitor for side effects of the medications.

### Statistical analysis

Paired t-tests were performed to assess response to treatments within each study group and unpaired t-tests were used to compare values between different treatment groups. Values at p < 0.05 were considered statistically significant.

## Results

**Fig. 3:**
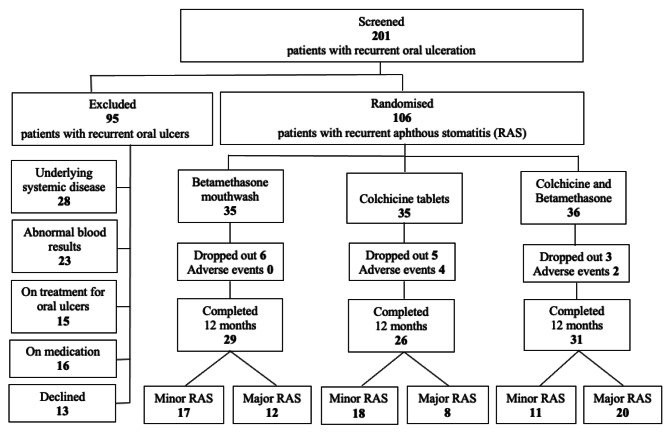
Diagram of the number of participants at each stage of the clinical trial. Patients screened for the clinical trial: diagnosis and treatment modality assigned and reasons for exclusion. Patients completing the clinical trial: diagnosis, treatment modality and incidence of side effects (201 screened, 106 randomised and 86 completed 12 months trial)

Of the 106 participants recruited to the trial, 86 completed the 12-month study period (39 males and 47 females). Of the 20 patients who did not complete, six patients developed adverse events, which necessitated termination of their medication and their participation in the study and 14 participants withdrew from the study due to time constraints, including both patients diagnosed with the herpetiform type of RAS. In the betamethasone group 29 out of 35 participants finished the 12 months trial (9 males and 20 females; 17 minor and 12 major RAS). In the colchicine group, 26 out of 35 completed the clinical trial (14 males and 12 females; 18 minor and 8 major RAS) and in the mixed therapy group 31 out of 36 completed the clinical trial (16 males and 15 females; 11 minor and 20 major RAS) (Fig. 3).

### Assessment of overall treatment efficacy

Of the 106 participants, six reported adverse reactions and all were on colchicine (gastric pain, peripheral neuropathy, and alopecia). The mean USS for all the participants was 34.9 ± 7.2 (SD) before treatment and 17.5 (± 8.9) at the end of the trial (p < 0.001). The mean USS progressively and significantly decreased after 3 months, 6 months and 9 months treatment (p < 0.001). Although there was a small further reduction in the mean USS after 12 months treatment, this was not statistically significant when compared with the score at 9 months but remained significantly less than the baseline visit (p < 0.0001) (Table 2). Out of the 74 patients, 86% showed significant clinical improvement by the end of the study and in 54 patients the USS fell to below 20 (63%).

**Table 2:** Comparison of the efficacy of three treatment modalities for recurrent aphthous stomatitis over one year

	USS range	Baseline USS	3 months USS	6 months USS	9 months USS	12 months USS
All patients (n = 86) (mean ± SD)	21–53	34.9 ± 7.2	24.5 ± 9.1*	21.1 ± 9.2*	18.8 ± 10.4*	17.5 ± 8.9
Betamethasone MW (n = 29) (mean ± SE)	22–52	34.6 ± 1.5	22.5 ± 1.5*	18.3 ± 1.5*	15.3 ± 1.8*	15.7 ± 1.7
**Colchicine Tabs (n = 26)** (mean ± SE)	21–50	33.0 ± 1.2	22.4 ± 2.0*	21.1 ± 1.9	20.9 ± 2.1	17.7 ± 1.9
**Colchicine Tabs + betamethasone MW (n = 31)** (mean ± SE)	25–53	36.9 ± 6.5	28.0 ± 1.3*	23.1 ± 8.9*	20.1 ± 1.8*	19.2 ± 1.4

USS = Ulcer Severity Score. Means and Standard Error of the Mean (SE) shown for treatment groups, and Standard Deviation (SD) for all patients. * P < 0.05 compared with previous visit. All values are p < 0.001 compared with baseline.

### Comparison between the efficacy of the three treatment modalities

**Fig. 4:**
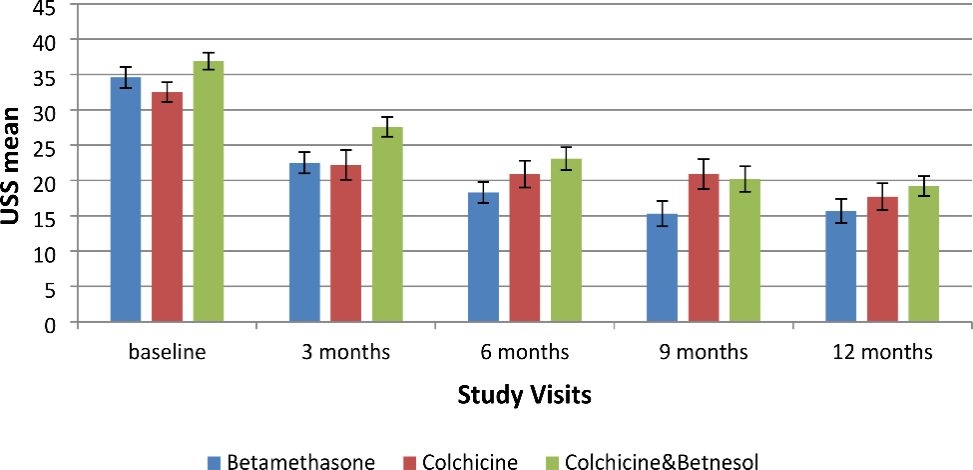
Sequential comparison of the mean Ulcer Severity Scores (± SE) in the three treatment groups. RAS patients (n = 86) followed for 12 months at 3-month intervals (a total of 5 visits). All mean values after treatment were significantly lower than baseline using paired t-test (p < 0.05)

The most effective clinical outcome (highest reduction in mean USS) after 12 months treatment was in the betamethasone group (mean 54.6% improvement) (Fig. 4). The USS decreased below 20 in 22/29 patients (76%) on betamethasone, 13/26 on colchicine (50%) and 19/31 (61%) on colchicine/betamethasone combination, suggesting that the regime with betamethasone solely was the most efficacious.

### The effect of treatment on different types of RAS

**Fig. 5:**
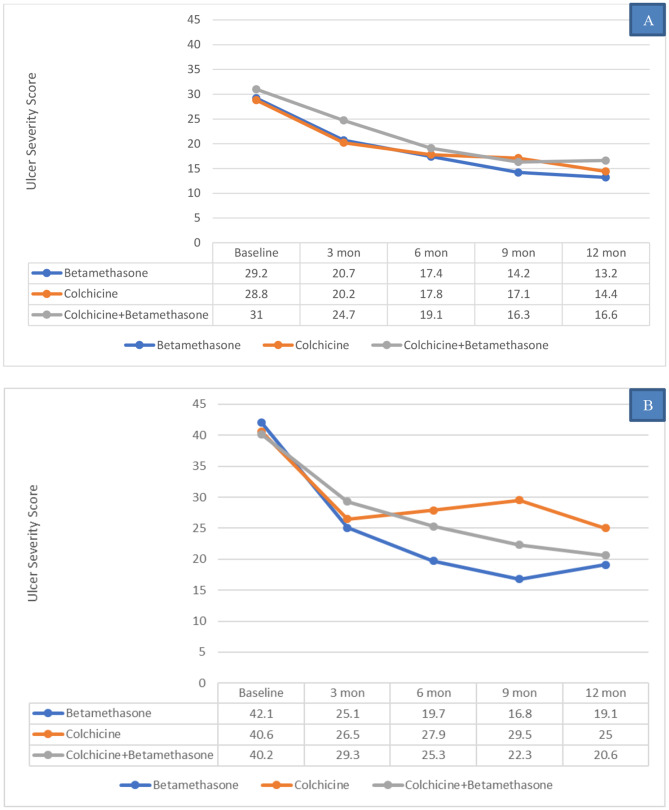
Comparison of the sequential response to treatment in the three study groups. **A**: Minor RAS. **B**: Major RAS

Of the 86 subjects who completed the clinical trial, 46 had minor and 40 had major RAS. The response to treatment in these two subgroups showed a similar trend to that noted in the series, with the mean USS progressively decreasing in both groups over 9 months but with no further significant improvement after 12 months (Fig. 5A and B). The percentage reduction in the USS compared with the baseline score was 50.8% for minor RAS and 48.5% for major RAS. Additionally, no gender differences were apparent. Overall, USS below 20 was achieved in 32 out of the 47 patients with minor RAS (68%) and 21 of 39 patients with major RAS (54%). However, this difference did not reach statistical significance.

Furthermore, variation was noted within the groups, as most patients showed a steady decline in ulcer severity over time, while others showed a rapid improvement but then relapsed. In most patients, little improvement was found beyond 6 months therapy. Moreover, two cases of minor RAS and two cases of major RAS appeared recalcitrant to treatment.

### Group 1 (Betamethasone Mouthwash)

#### Minor RAS

in this group, there were 17 minor RAS subjects, with mean USS score prior treatment of 29.2 ± 3.9 (range 22–36). After 3 months therapy, the USS improved by 29.2% to a mean of 20.7 (p < 0.0001) (Table 2). Further significant improvement was found at 6 and 9 months (p < 0.01). Further reduction of the USS after 12 months therapy was not statistically significant, compared with previous visit score (Table 3).

#### Major RAS

The mean USS score before treatment of 12 major RAS subjects was 42.1 ± 5.5 (range 35–53), and this decreased significantly after 3 and 6 months of treatment (p < 0.01) (Tables 2 and 3). Little further improvement was found after 6 months treatment, and the USS means at 9 and 12 months were not significantly reduced compared with the 6 months visit. Additionally, all patients with major RAS showed reduction in the USS after 6 months’ therapy and little further improvement beyond this, suggesting that 6 months might be optimal for evaluating the efficacy of treatment in Major RAS.

**Table 3:** Comparison of responses of patients with major or minor RAS to three different therapy regimes over twelve months (USS mean ± SD).

Study Group	Type of Ulcer	Baseline USS (range)	3-months USS	6 months USS	9 months USS	12 months USS
**Betamethasone Mouthwash**	**Minor** **(n = 17)**	29.2 ± 3.9 (22–36)	20.7 ± 7.3*	17.4 ± 8.0*	14.2 ± 9.7*	13.2 ± 9.4
	**Major** **(n = 12)**	42.1 ± 5.5 (35–53)	25.1 ± 8.9*	19.7 ± 8.8*	16.8 ± 9.7	19.1 ± 8.4
**Colchicine Tablets**	**Minor** **(n = 18)**	28.8 ± 4.4 (20–37)	20.2 ± 8.9*	17.8 ± 9.6	17.1 ± 9.3	14.4 ± 8.7
	**Major** **(n = 8)**	40.6 ± 4.1 (35–48)	26.5 ± 13.8*	27.9 ± 6.9	29.5 ± 8.9	25.0 ± 8.1
**Colchicine Tablets plus Betamethasone Mouthwash**	**Minor** **(n = 11)**	31.0 ± 3.3 (25–36)	24.7 ± 3.8*	19.1 ± 8.5*	16.3 ± 10.6	16.6 ± 7.2
	**Major** **(n = 20)**	40.2 ± 5.5 (33–53)	29.3 ± 8.9*	25.3 ± 8.5*	22.3 ± 9.8*	20.6 ± 7.9

*Indicates statistically significant decrease compared with the score on the previous visit (p < 0.05). USS = ulcer severity score

### Group 2 (Systemic Colchicine)

#### Minor RAS

the mean baseline of USS before treatment of 18 patients with minor RAS was 28.8 ± 4.4, and this was reduced by almost 30% after 3 months treatment to 20.2 ± 8.9 (p < 0.001). The improvement in the USS continued incrementally during the study period. At the end of the study, the mean USS had improved by more than 50% when compared with the baseline (p < 0.001) (Table 3) and the USS reduced to below 20 in 12 of the 18 patients (66.6%).

#### Major RAS

the mean USS of the 8 subjects in this group was 40.6 ± 4.1 (range 35–68). After 3 months therapy the mean USS improved by 34.8% (p < 0.02). Further improvement in this group was found after 3 months, suggesting that 3 months therapy might be the minimal period to assess the efficacy of treatments (Table 3). The USS reduced to below 20 in 1 of the 8 patients (13%).

### Group 3 (systemic colchicine plus Betamethasone Mouthwash)

#### Minor RAS

the mean USS of the 11 minor RAS subjects before treatment was 31 ± 3.3 (range of 25–36). After 3 months treatment, there was significant improvement of the USS by 20.3% with a further significant reduction in the mean USS at 6 months (p < 0.001), but no further statistically significant improvements thereafter (Table 3). The USS reduced to below 20 in 8 of the 12 patients (67%).

#### Major RAS

the mean USS of the 20 major RAS subjects before treatment was 40.2 ± 5.5 (range 33–53), and this was significantly reduced after 3 months treatment by 27.1% (p < 0.0002). The scores continued to decrease significantly after 6 and 9 months therapy (p < 0.03), but then stabilised. At the end of the 12 months therapy period, there was 48.7% improvement in the mean USS compared with the baseline visit (p < 0.001) (Table 3). The USS reduced to below 20 in 11 out of the 19 patients (58%).

Analysis of the results in terms of the USS reduction below 20 showed that the overall response to treatment for minor RAS (32/47, 68%) was greater than for major RAS (21/39, 54%) but was not significantly different. There was substantial improvement in the USS after 6 months treatment in all three groups, and while the mean USS continued to reduce in the next 6 months of treatment, the benefit was statistically insignificant. Moreover, the greatest reduction in the mean USS after 12 months treatment was in the betamethasone mouthwash group for both minor and major RAS.

## Discussion

This clinical trial has provided formal clinical evidence for the efficacy of betamethasone mouthwash in the treatment of minor and major RAS. This trial has demonstrated that colchicine tablets alone or in combination with betamethasone mouthwash was an effective treatment, although not significantly better than a regime with betamethasone mouthwash alone. This study has also shown that optimal improvement is perceived in the first three months of treatment but that for betamethasone mouthwash, at least six months of continuous treatment is required for optimal improvement in ulcer severity.

One novel aspect of this study was to undertake a longer-term trial to address the question of how long a treatment regime was needed to induce a more sustained reduction in ulcer severity. This meant that it was deemed ethically inappropriate to include a non-treatment group, even though there are studies which have shown a significant effect of placebo in the management of RAS [16, 17]. Further review of patients after the period of active treatment would have allowed assessment of the longer-term treatment effects. This was not part of the protocol in this study but can be recommended for future studies. Furthermore, this trial showed that patients who did not respond to treatment in the first three months (14% irrespective of age or gender), were highly unlikely to do so subsequently. It is therefore recommended that in unresponsive RAS patients, the medication should be changed after three months of treatment.

The effectiveness of colchicine in the management of RAS has been reported in the literature [4, 10], but there is no comparison of its efficacy with other therapies nor long term sequential assessment to determine the optimum therapy duration or dose. Our findings showed that colchicine at a low dose of 500 mcg a day had a significant effect especially on minor RAS over the first 3 months of therapy only, with little further improvement seen thereafter, except in major RAS in combination with betamethasone mouthwash. This relatively low dose of colchicine was chosen to minimise side effects previously reported with higher doses. In a study by Oh et al. (2022) [18], 22.8% of the study group who were taking colchicine 1.2 mg/day experienced side effects within 2 weeks of starting the treatment, which resulted in its discontinuation. Interestingly, a reduction from 1.2 mg/day to 300–600 mcg/day reduced the side effects in more than a third of the patients. Therefore, precautions were taken into consideration when designing the current study, and thus, a dose of 500 mcg/day was deemed to be the optimum therapeutic dose. However, our data also suggest that in cases where colchicine 500 mcg daily is not effective after three months, either the dose can be increased to 1000 mcg or an alternative therapy should be considered.

Regarding adverse effects, Six of the 106 participants reported adverse reactions, and all were on colchicine. Adverse effects of colchicine have been reported in previous studies [13, 19]. The benefits anticipated to be gained from prescribing colchicine (with or without betamethasone) have therefore to be balanced against its potential side effects.

The results of the present study showed that topical betamethasone, systemic colchicine or a combined therapy of colchicine 500 mcg per day plus betamethasone mouthwash all resulted in significant benefits within the first three months of therapy. However, with topical treatment, the maximum reduction in mean ulcer scores was reached by nine months therapy. This suggests that this therapy could be sustained for up to 9 months for maximum effect and without detectable adverse reactions. Further reduction in the USS with dual therapy continued for 6 months in the minor RAS group and at 9 months in the major RAS group, suggesting that more sustained therapy might be needed for effective responses in major RAS. This supports a perception that for the management of the severe and constantly recurring ulcers, topical treatment may not be enough and systemic medications can be added. However, our results suggest that the added benefit of taking colchicine 500 mcg tablet daily was limited and possibly increasing the dose, in line with its use medically, may be more effective.

One of the limitations in the present study is the lack of a placebo controlled group, which was due to the study’s long timespan of 12 months and the fact that patients with severe types of RAS in particular could not be left without treatment for the long period of the clinical trial. Another limitation of this study is the lack of blinding in clinicians and participants, which was due to the.

differences in the mode of the applications since one is a tablet and the other is a mouthwash.

In conclusion, topical betamethasone mouthwash was shown to be an effective treatment for minor and major RAS subtypes and this study provided evidence for its efficacy as a first-line treatment in RAS. The effect of therapy on RAS may be incremental and the optimal duration of treatment was six months.

## Data Availability

All data generated or analysed during this study are included in the original thesis and is present in the related manuscript files.

## List of Abbreviations

RAS: Recurrent Aphthous Stomatitis
USS: Ulcer Severity Score
PFAPA: Periodic fever, Aphthous stomatitis, Pharyngitis, Cervical adenitis
ICH-GCP: International Council for Harmonisation- Good Clinical Practice
ISRCTN: The International Traditional Medicine Clinical Trial Registry
SE: Standard error of the mean
