# Correction: Topical betamethasone and systemic colchicine for treatment of recurrent aphthous stomatitis: a randomised clinical trial

**DOI:** 10.1186/s12903-023-03634-3

**Published:** 2023-12-08

**Authors:** Surab Alsahaf, Khlood A. Alkurdi, Stephen J. Challacombe, Anwar Tappuni

**Affiliations:** 1 Oral Medicine, Centre for Host Microbiome Interactions, Faculty of Dentistry, Oral & Craniofacial Sciences, King’s College London, and Guys and St Thomas’ NHS Foundation Trust, London, UK; 2 Institute of Dentistry, Faculty of Medicine and Dentistry, Queen Mary University of London, Office 7, Floor 4, London, E1 2AD UK

**Correction to: BMC Oral Health (2023) 23:709**

10.1186/s12903-023-03335-x.

In the Abstract section of the article [1], there is an error in the ISRCTN ID. The correct ID should read ISRCTN32677164.

